# Possible scenarios for occurrence of M ~ 7 interplate earthquakes prior to and following the 2011 Tohoku-Oki earthquake based on numerical simulation

**DOI:** 10.1038/srep25704

**Published:** 2016-05-10

**Authors:** Ryoko Nakata, Takane Hori, Mamoru Hyodo, Keisuke Ariyoshi

**Affiliations:** 1 Research and Development Center for Earthquake and Tsunami, Japan Agency for Marine-Earth Science and Technology, 3173-25, Showa-machi, Kanazawa-ku, Yokohama, Kanagawa 236-0001, Japan

## Abstract

We show possible scenarios for the occurrence of M ~ 7 interplate earthquakes prior to and following the M ~ 9 earthquake along the Japan Trench, such as the 2011 Tohoku-Oki earthquake. One such M ~ 7 earthquake is so-called the Miyagi-ken-Oki earthquake, for which we conducted numerical simulations of earthquake generation cycles by using realistic three-dimensional (3D) geometry of the subducting Pacific Plate. In a number of scenarios, the time interval between the M ~ 9 earthquake and the subsequent Miyagi-ken-Oki earthquake was equal to or shorter than the average recurrence interval during the later stage of the M ~ 9 earthquake cycle. The scenarios successfully reproduced important characteristics such as the recurrence of M ~ 7 earthquakes, coseismic slip distribution, afterslip distribution, the largest foreshock, and the largest aftershock of the 2011 earthquake. Thus, these results suggest that we should prepare for future M ~ 7 earthquakes in the Miyagi-ken-Oki segment even though this segment recently experienced large coseismic slip in 2011.

A Mj9.0 great interplate earthquake occurred off the east coast of Tohoku district, northeast Japan, on March 11, 2011, with ruptures propagating over a distance of 400 km along the Japan Trench^1^. In the past, in response to subduction of the Pacific Plate beneath northeast Japan, M ~ 7 interplate earthquakes have occurred repeatedly in multiple segments along the Japan Trench (Fig. 1). Some source areas of these earthquakes have ruptured as a result of foreshock or coseismic slip or aftershock of the 2011 Mj9.0 earthquake.

The deeper part of the Mj9.0 coseismic slip area is located off the east coast of Miyagi Prefecture, where Mj7.1–7.4 earthquakes have occurred with recurrence intervals of approximately 30–40 years, in 1897, 1936, 1978, and 2005^2^. The rupture areas of these earthquakes, referred to as Miyagi-ken-Oki earthquakes, partly overlapped, and their maximum seismic slips were less than 3 m^3^,^4^. The source regions of past Miyagi-ken-Oki earthquakes ruptured again during the Mj9.0 earthquake, with coseismic slip in the Miyagi-ken-Oki segment estimated to be approximately 5–15 m^1^,^5^,^6^,^7^,^8^,^9^. These slip amounts are larger than that of past Miyagi-ken-Oki earthquakes.

The southern segments to the Miyagi-ken-Oki segment are known as Fukushima-ken-Oki and Ibaraki-ken-Oki segments^2^ (Fig. 1). Coseismic ruptures have propagated to the Fukushima-ken-Oki segment. Three Mj7.3–7.5 earthquakes occurred sequentially in November 1938 in this segment, and M < 7 earthquakes have occurred repeatedly afterward. Recently, Mj6.9 and Mj6.7 earthquakes occurred in July 2008 and March 2010, respectively. The largest aftershock of the Mj9.0 earthquake, a Mj7.6 event, was recorded 29 min following the mainshock in the Ibaraki-ken-Oki segment. In this segment, M ≤ 7 earthquakes have occurred repeatedly with recurrence intervals of 20–30 years, including a Mj7.0 earthquake in May 2008.

One of the characteristics of the 2011 earthquake is the sequence of seismic and aseismic events observed within the Mj9.0 source area prior to the mainshock. On March 9, 2011, 51 h prior to the Mj9.0 earthquake, a Mj7.3 interplate earthquake occurred within the Mj9.0 source area^6^,^10^. This event was the largest foreshock of the Mj9.0 earthquake, and its afterslip of the foreshock propagated to the surrounding area and triggered the Mj9.0 dynamic rupture on March 11, 2011^11^. A Mw7.1 earthquake occurred in January 1981^3^ near the foreshock area, and a Mw6.9 earthquake occurred in October 2003^12^ near the rupture initiation area. In addition, two slow slip events (SSEs) were observed in 2008 and 2011 within the coseismic slip area of the 2011 earthquake^13^. The second SSE occurred in February 2011 and continued at least until the occurrence of the largest foreshock^13^.

More than 5 years have passed since the Mj9.0 earthquake. The post-seismic deformation rate is decreasing, and it has almost decayed by the time 2.5 years following the mainshock^14^. The afterslip resulting from the Mj9.0 earthquake at a deeper area than the coseismic slip area was estimated to be approximately 2.5 m during a 220-day period^15^, and more than 2–4 m during the 2.5-year^14^ period. The afterslip was smaller than that occurring near the source area of past Miyagi-ken-Oki earthquakes. Moreover, the M > 7 Miyagi-ken-Oki earthquake had not yet occurred. If we consider only cumulative slip within the source region of past Miyagi-ken-Oki earthquakes, we may expect that the Miyagi-ken-Oki earthquake will not occur for at least the past recurrence interval. However, large coseismic slip extending over a wide region including the Miyagi-ken-Oki area occurred during the 2011 Mj9.0 earthquake. Therefore, spatiotemporal development of stress accumulation and release as preparation process of the next Miyagi-ken-Oki earthquake may be different from that of past observations.

In the Tohoku-Oki region, earthquakes having a similar magnitude as the 2011 earthquake occurred in 869 in the Jogan era and 1454 in the Kyotoku era as indicated by geological data of tsunami sediments^16^,^17^,^18^, and historical documents^19^. Therefore, such evidence indicates that the recurrence interval of these great earthquakes is 500–1000 years.

Previous numerical simulations of the earthquake generation cycles of the 2011 Tohoku-Oki earthquake have shown that M ~ 9 earthquakes have occurred with a recurrence interval of 700–900 years^20^,^21^,^22^. However, such studies differ significantly on the occurrence time of the next (immediately following the Tohoku earthquake) Miyagi-ken-Oki earthquake. Numerical simulations of a two-dimensional (2D) model performed by using a composite law^23^, which is a type of rate- and state-dependent friction law, showed that two smaller earthquakes than that occurring at a shallower part will occur at a deeper part within 10 years following the mainshock^20^. The first was followed by the mainshock with a typical delay of 196 days. However, such an earthquake has not been observed at present. In contrast, Ohtani *et al.*^22^ used a hierarchical asperity model with a three-dimensional (3D) plate interface and showed that no M ~ 7 earthquake will occur in the Miyagi-ken-Oki region during more than 150 years following the M ~ 9 earthquake^22^. A 3D model by Shibazaki *et al.*^21^ used a different type of rate- and state-dependent friction law with two state variables and showed that no significant slip events will occur during 150 years following megathrust events. However, neither simulation using a 3D model could reproduce the southern limit of the coseismic slip area and the largest aftershock in the Ibaraki-ken-Oki segment. In addition, Kato and Yoshida^20^ and Ohtani *et al.*^22^ showed that a foreshock will occur 2.2 days and 62 days prior to the M ~ 9 earthquake, respectively. However, no foreshock was observed in the simulation performed by Shibazaki *et al.*^21^.

In this study, we reproduce the observational characteristics prior to, during, and following the 2011 Mj9.0 earthquake including past Miyagi-ken-Oki earthquakes and foreshock, mainshock, largest aftershock, and afterslip by using realistic numerical simulation. We discuss the time interval between the Miyagi-ken-Oki earthquake immediately following the M ~ 9 earthquake and the M ~ 9 earthquake by using reasonable scenarios consisting of various observational characteristics. In addition, we investigate the scenario prior to the next M ~ 9 earthquake.

The numerical simulations are based on the method used in previous studies^22^,^24^, and shown in Methods. We modified the distribution of frictional parameters from the hierarchical asperity model^22^. In addition, we used five circular patches termed SHL1, SHL2, MYG, FKS, and IBK as the past sources of the 1981, 2003, 1978 Miyagi-ken-Oki, and 1938 Fukushima-ken-Oki earthquakes, and the 2011 Ibaraki-ken-Oki largest aftershock, respectively (Fig. 2). Although the rupture areas of past Miyagi-ken-Oki earthquakes were not completely overlapped, we simply modeled the repeating M > 7 earthquakes at the Miyagi-ken-Oki segment by using a circular patch.

## Results

**Figure 1:**
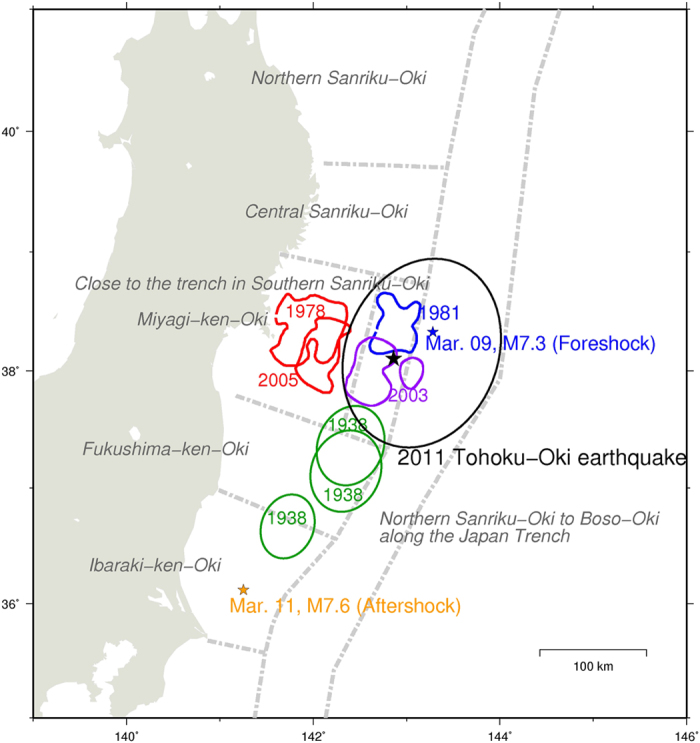
Distribution of the source areas of past large earthquakes along the Japan Trench. The large slip area of the 2011 Tohoku-Oki earthquake is approximated by the black ellipse^1^,^5^,^6^,^7^. The black, blue, and orange stars indicate the hypocenters of the mainshock, foreshock, and the largest aftershock of the 2011 events, respectively. The blue and purple areas indicate the source region of the 1981^3^ and 2003^12^ earthquakes, respectively. The red areas indicate the source areas of the 1978 and 2005 Miyagi-ken-Oki earthquakes^3^,^4^. The three green ellipses approximate the source areas of the November 1938 Fukushima-ken-Oki earthquakes^36^. The gray dotted lines and italic characters indicate segmentations^2^. The map was created by using Generic Mapping Tools software (GMT v4.5.12; http://gmt.soest.hawaii.edu/)^37^.

**Figure 2:**
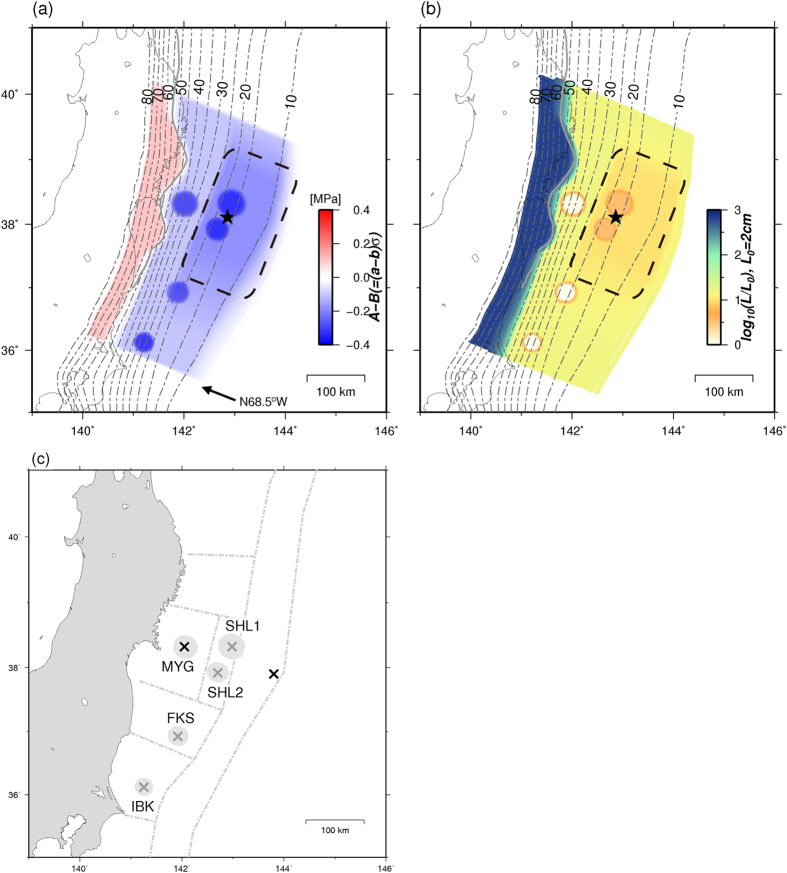
Distribution of frictional parameters. (**a**) Spatial distribution of (A–B) (MPa). The dashed-line rectangle indicates the frictional heterogeneity area for the M ~ 9 earthquake assumed in this model. Contours indicate depth (km) to the upper surface of the descending plate^31^. The gray solid line indicates the western limit of interplate earthquake distribution^38^. The black star shows the hypocenter of the 2011 Tohoku-Oki earthquake. (**b**) Characteristic slip distance (L). (**c**) Distribution of the source areas of M ~ 7 earthquakes during the interseismic period of the Mw9.1 earthquake resulting from the simulations. Crosses indicate points shown in Figs 3 and 4. The maps were created by using Generic Mapping Tools software (GMT v4.5.12; http://gmt.soest.hawaii.edu/)^37^.

We performed approximately 190 simulation runs by using various parameter sets. From the various simulations performed, we qualitatively reproduced the observational characteristics by using several frictional parameter sets, as shown in Fig. 2a,b and in Table 1, S1, and S2. The frictional heterogeneity at the shallower part of the Miyagi-ken-Oki segment, which is close to the trench in the southern Sanriku-Oki segment and northern Sanriku-Oki to Boso-Oki along the Japan Trench segment described in Fig. 1, was assumed to be slightly stronger than that in the surrounding area over a wide range (Methods). In contrast, Ohtani *et al.*^22^ assumed a very strong heterogeneity of (A–B) at the large coseismic slip area. The value of (A–B) was eight times larger than that of the surrounding area in their study. In addition, we used a small value of seismic radiation damping term (η = 0.3G/2β)^25^ to reproduce a shorter duration during the 2011 Mj9.0 earthquake. Here, we show the various results obtained with different sets of frictional parameters shown in Table 1, S1,S2. Each result has similar characteristics that are consistent with observations. As detailed below, we describe these characteristics by dividing a M ~ 9 earthquake cycle into three periods such as that immediately following the M ~ 9 earthquake, an interseismic period, and a later stage.

### M ~ 9 earthquake, M ~ 7 aftershock, and afterslip of M ~ 9 earthquake

**Figure 3:**
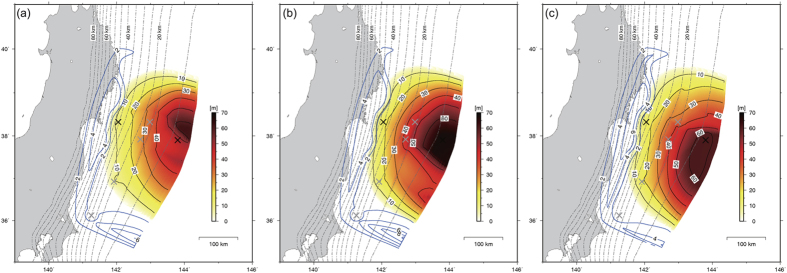
Coseismic slip distribution of the Mw9.1–9.2 earthquake when V > 1.0 cm/s. The blue contours indicate afterslips for approximately three years since each earthquake. (**a**) Same events as those shown in Fig. 5c–e. The parameter set shown in Table 1 was used. (**b**) Same events as those shown in Fig. S1a–c. The parameter set shown in Table S1 was used. (**c**) A different event using the parameter sets in Table S2. The maps were created by using Generic Mapping Tools software (GMT v4.5.12; http://gmt.soest.hawaii.edu/)^37^.

**Figure 4:**
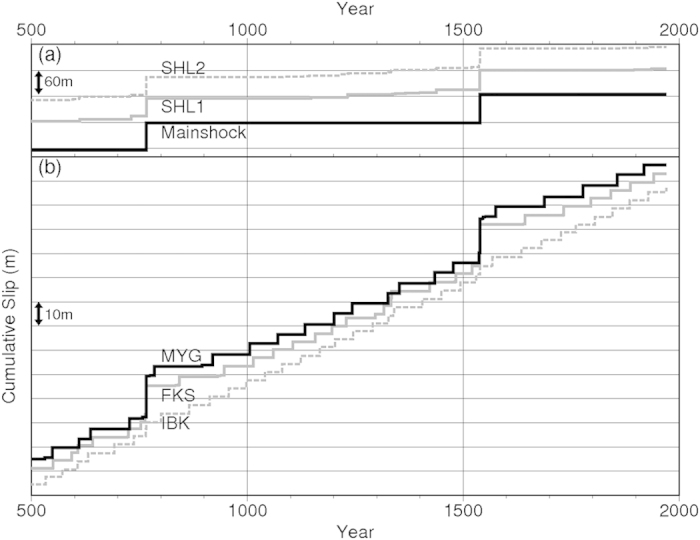
Temporal variation of cumulative slip obtained by using the parameter set listed in Table 1. (**a**) Crosses within the M ~ 9 slip area, SHL1, and SHL2 represented in Figs 2 and 3. (**b**) Crosses in MYG, FKS, and IBK patches represented in Figs 2 and 3.

In each parameter set, two Mw9.1–9.2 earthquakes occurred with recurrence intervals of approximately 700–770 years (Figs 3 and 4a). The maximum coseismic slips of these earthquakes were 58–68 m near the trench (Fig. 3). Although the spatial distributions of large coseismic slip areas >30 m differed slightly from each other, the contours of the 10-m-slip were almost similar among these models. The coseismic slips in the MYG and FKS patches during each M ~ 9 earthquake were approximately 10–20 m (Fig. 3), whereas no coseismic slips were observed in the IBK patch during each M ~ 9 earthquake (Fig. 3).

The rupture initiation point differed among M ~ 9 earthquakes, occurring in the southern area near the SHL2 patch, the northern area near the SHL1 patch, and the shallower area. The M ~ 9 earthquake initiated from a point within the large coseismic slip area of each earthquake.

A Mw7.0–7.2 aftershock occurred in the IBK patch within 2–50 days following each M ~ 9 earthquake. Furthermore, the afterslip following the M ~ 9 earthquake was distributed in the deeper side of the coseismic slip area. In addition, the afterslip in the MYG patch was less than 2 m during approximately three years (Fig. 3). When we set the value of L (e.g., 0.6 m, 0.8 m, or 1.0 m) at the background area (A–B < 0) to be larger than that in Table 1 (0.3 m), afterslip also occurred in the north area of the coseismic slip area of the M ~ 9 earthquake.

### M ~ 7 earthquakes during interseismic periods of M ~ 9 earthquakes

Mw7.1–7.4 earthquakes occurred repeatedly in the MYG patch during interseismic periods of M ~ 9 earthquakes (Figs 2c and 4b) with maximum coseismic slips of 3–5 m. In the case of the parameter set shown in Fig. 2a,b, the recurrence interval of five earthquakes in the MYG patch during 230 years prior to the Mw9.1 earthquake, shown in Fig. 3a, was 26–91 years at an average of 54 years and standard deviation of 24.5 years (Fig. 4). Similar to the scenarios mentioned above, the M ~ 9 earthquake occurred 0.1–50 years following the latest MYG earthquake.

During the same 230-year period, Mw6.9–7.1 earthquakes occurred repeatedly in the IBK patch with average recurrence intervals of 39 years at a standard deviation of 12.3 years (Fig. 4b). In the FKS patch, Mw6.7–7.1 earthquakes occurred repeatedly with average recurrence intervals of 36 years and a standard deviation of 24.2 years (Fig. 4b).

During the last 200 years of the M ~ 9 earthquake cycle resulting from 19 simulations with five M ~ 7 patches (Fig. 2c), M ~ 7 earthquakes occurred repeatedly with average recurrence intervals of 54, 36, and 40 years and standard deviations of 19.5, 26.6, and 11.4 years in the MYG, FKS, and IBK patches, respectively.

In the SHL1 patch, two or three Mw7.2–7.4 earthquakes occurred during the interseismic period of the two M ~ 9 earthquakes, depending on the model (Fig. 4a). In the SHL2 patch, no or one Mw7.0–7.1 earthquake occurred during that period (Fig. 4a). The M ~ 7 earthquake such as in 2003 was not occurred at the SHL2 patch in our trial on frictional parameter sets.

**Figure 5:**
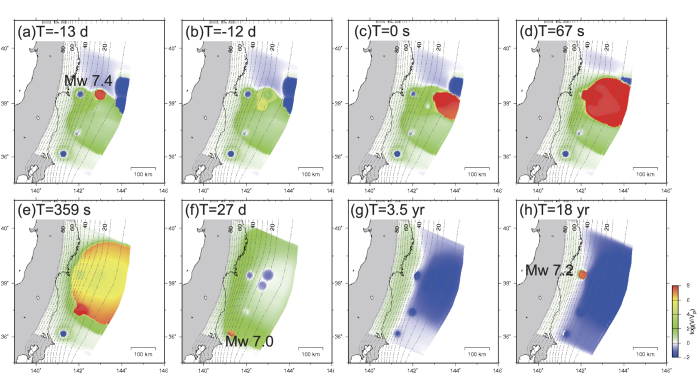
Slip velocity distribution normalized to the plate convergence rate from the occurrence of foreshock of the Mw9.1 earthquake to 18 years following the Mw9.1 earthquake. Blue and red areas indicate locked and unstably slipping parts of the fault, respectively. Yellow/green and white indicate slow slip and plate convergence rates, respectively. (**a**) Occurrence of Mw7.4 earthquake (foreshock) in the SHL1 patch. (**b**) Acceleration of slip in the SHL2 patch. (**c**) Initiation of Mw9.1 earthquake. (**d**) Propagation of coseismic rupture of the Mw9.1 earthquake to the MYG patch. (**e**) Propagation of coseismic rupture of the Mw9.1 earthquake to the FKS patch. (**f**) Occurrence of Mw7.0 earthquake (aftershock) in the IBK patch. (**g**) Continued afterslip. (**h**) Occurrence of Mw7.2 earthquake in the MYG patch. The maps were created by using Generic Mapping Tools software (GMT v4.5.12; http://gmt.soest.hawaii.edu/)^37^.

At the later stage of the interseismic periods of the M ~ 9 earthquake cycle, the locked state remained at the shallowest area. Moreover, transient slow slips occurred near the rupture initiation area at the end of each M ~ 9 earthquake cycle (Fig. 5a,b). These slips were generated by the afterslip of any one M ~ 7 earthquake in the SHL1, MYG, FKS, and IBK patches. Occasionally, one of these slow slips appeared to trigger a M ~ 9 earthquake. These transient slips seemed to be comparable to that produced by the previous simulation study^22^. Moreover, one of these transient slow slips, occurred prior to the M ~ 9 earthquake likely to be comparable to those reported in several geodetic studies^26^,^27^,^28^.

**Figure 6:**
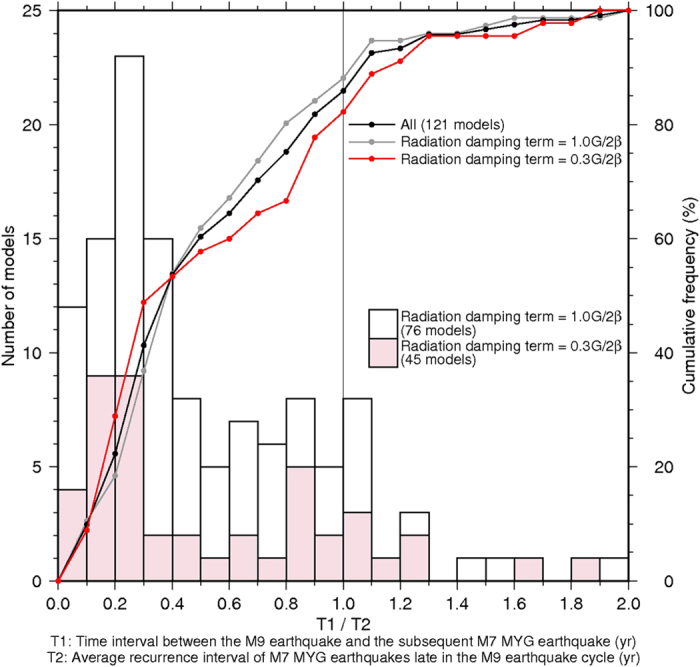
Distribution of the ratio of T1 and T2. T1 is the time interval between the M ~ 9 earthquake and the subsequent M ~ 7 MYG earthquake. T2 is the average recurrence interval of M ~ 7 MYG earthquakes late in the M ~ 9 earthquake cycle. The red bars with the left vertical axis and the red polygonal line with the right vertical axis represent the number and cumulative frequency (%) of the simulations using a small value of radiation damping term (η = 0.3G/2β) to reproduce a shorter duration during the M ~ 9 earthquake. Step size of the dots on the line graph are 0.1, which is the same as that of the bar graph. The open bars and the gray polygonal line show the results using a large value of radiation damping term (η = 1.0G/2β). The black polygonal line show the results from both red and open bars. The average recurrence interval of M ~ 7 MYG earthquakes were calculated during 200 years before the M ~ 9 earthquake. Results that could not reproduce Mw > 8.5 earthquakes or the repeating M ~ 7 MYG earthquakes were not included. Therefore, total 121 models are shown in this figure.

The above characteristics were observed in many simulations using other frictional parameter sets such as those shown in Table S1, S2 based on Fig. 2 and Table 1, and they correspond approximately to the observed characteristics prior to, during, and following the 2011 Tohoku-Oki earthquake. In 86% of the reasonable scenarios, the Mw7.1–7.3 earthquake spontaneously occurred within 100 years (e.g., 18 years in Fig. 5h) following the M ~ 9 earthquake in the MYG patch, in which the time interval was equal to or shorter than the past recurrence interval in the later stage of the M ~ 9 earthquake cycle (Fig. 6).

### M ~ 7 foreshock and M ~ 9 mainshock

The timing of the M ~ 7 foreshock differed for each cycle. One model, shown in Fig. 2 and Table 1, showed a scenario in which a Mw7.4 earthquake occurred in the SHL1 patch 13 days prior to the Mw9.1 simulated earthquake. This event corresponded to the foreshock occurring on March 9 (Fig. 4a, gray solid line; Fig. 5a). Moreover, the afterslip of the foreshock propagated toward the south and appeared to trigger the Mw9.1 earthquake (Fig. 5b,c). Moreover, the Mw9.1 coseismic rupture propagated to the MYG patch approximately 1 min following its initiation (Fig. 5d). These results are similar to the observation during the 2011 earthquake^6^,^11^. However, in this model, the rupture initiated at the shallower area near the SHL1 patch, and the rupture propagation was delayed in the FKS patch (Fig. 5c,e).

A different model (Table S1) showed a scenario in which no earthquake occurred as a foreshock in the SHL1 patch prior to the Mw9.2 earthquake (Fig. S1). The coseismic rupture of the Mw9.2 earthquake initiated near the SHL2 patch (Fig. S1a). The MYG and FKS patches ruptured approximately 90 s and 110 s following the rupture initiation, respectively (Fig. S1b,c). During the 2011 M ~ 9 earthquake, the 1978 Miyagi-ken-Oki and 1938 Fukushima-ken-Oki source areas were ruptured approximately 20–90 s and 100–160 s following the rupture initiation^1^,^6^. Except for the non-occurrence of a M ~ 7 foreshock, the rupture initiation area and propagation time in this scenario were very similar to the observation during the 2011 earthquake.

Although we reproduced various characteristics, the M ~ 7 foreshock in the SHL1 patch that occurred 51 h prior to the Mj9.0 earthquake could not be reproduced by all models. In 15% and 30% of the simulations, the M ~ 7 foreshock in the SHL1 patch occurred within 4 days and 50 days prior to the M ~ 9 earthquake, respectively. For the simulations, parameter sets with a small radiation damping term of 0.3G/2β were used as those shown by red color in Fig. 6. These results suggest that the foreshock that occurred a few days earlier within the source area of the M ~ 9 earthquake, such as the Mj7.3 earthquake occurring on March 9, 2011, was not a necessary condition for the occurrence of the M ~ 9 earthquake along the Japan Trench. It should be noted that we did not detect a relationship between the occurrence time of the M ~ 7 foreshock in the SHL1 patch and the rupture initiation area of the M ~ 9 earthquake.

## Discussion

We reproduced the observational characteristics of the recurrence interval, coseismic slip, aftershock, and afterslip of the 2011 Mj9.0 earthquake and the recurrence interval of M ~ 7 earthquakes (MYG, FKS, and IBK) along the Japan Trench. A range in recurrence intervals of M ~ 7 earthquakes was detected at the MYG, FKS, and IBK patches depending on slight differences in frictional parameters. Compared with past observations, the range of recurrence interval simulated in this study is considered to be acceptable.

In this study, we generally reproduced coseismic slips of both M ~ 7 and M ~ 9 earthquakes in the Miyagi-ken-Oki segment with a simple model by using a circular patch. Similar to the observations of the 2011 Mj9.0 earthquake, a larger coseismic slip was observed in the MYG patch during the M ~ 9 earthquake than that in the M ~ 7 MYG earthquake. Nonetheless, the results showed that the time interval between the M ~ 9 earthquake and the subsequent MYG earthquake was shorter than the average recurrence interval observed in the later stage of the M ~ 9 earthquake cycle (Fig. 6). Long-term quiescence was not expected in the MYG patch following the M ~ 9 earthquake. The earlier occurrence of M ~ 7 earthquakes at the deeper part is similar to the results of Kato and Yoshida^20^. We describe the scenario more specifically and practically in each deeper segment of the Miyagi-ken-Oki, Fukushima-ken-Oki, and Ibaraki-ken-Oki segments following the reproduction of important characteristics.

**Figure 7:**
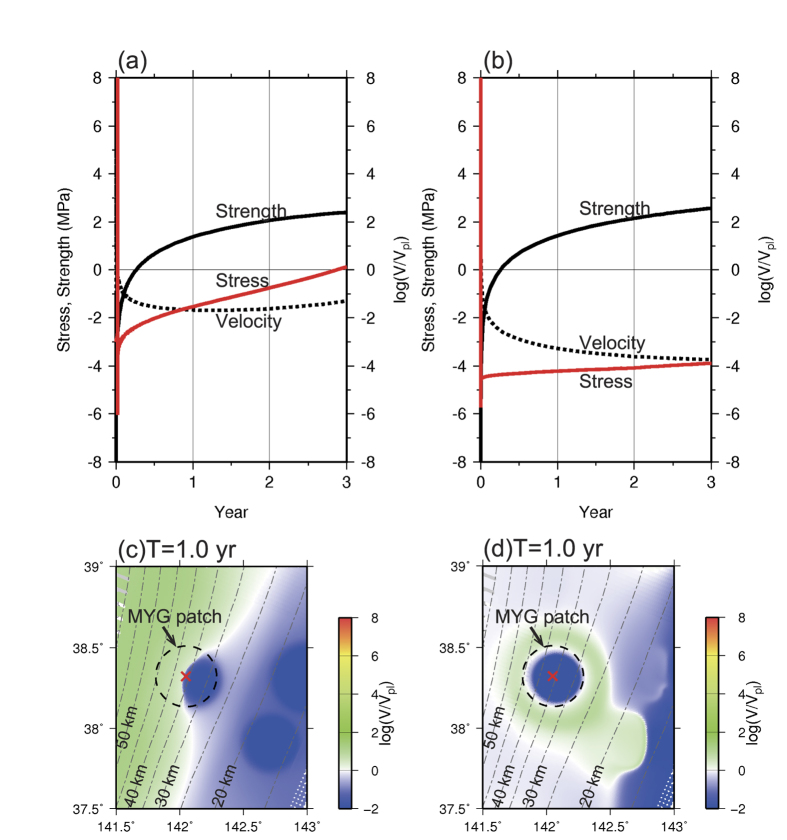
(**a**) Strength (solid black line), slip velocity (dotted black line), and stress (solid red line) over a 3 year period immediately following the Mw9.1 earthquake shown in Figs 3a and 5c–e. (**b**) The strength, slip velocity, and stress over a 3 year period following the M ~ 7 MYG earthquake during the later stage of the Mw9.1 earthquake cycle. (**c**) The slip velocity distribution normalized to the plate convergence rate near the MYG patch at the time of T = 1.0 year in Fig. 7a. The red cross indicates the point shown in Fig. 7a. (**d**) Slip velocity distribution normalized to the plate convergence rate near the MYG patch at the time of T = 1.0 year in Fig. 7b. The red cross indicates the point shown in Fig. 7b. The maps were created by using Generic Mapping Tools software (GMT v4.5.12; http://gmt.soest.hawaii.edu/)^37^.

To understand the shorter time interval of the MYG earthquake immediately following the M ~ 9 earthquake (Fig. 6), we compared the strength, stress, and slip velocity over a 3 year period at a point within the MYG patch (red cross in Fig. 7c,d) during two periods obtained from the same model (Fig. 7a,b). Following the Mw9.1 earthquake (Figs 3a and 5c–e), the stress increased at a high rate (solid red line in Fig. 7a) and the center of the MYG patch was located on the boundary between the afterslip area on the deeper side of the MYG patch and the locked area on the shallower side of the that patch (Fig. 7c). The high stress rate at the center of the MYG patch continued throughout the afterslip period. The rapid loading caused by the significant afterslip of the great earthquake was also simulated in the 2D model^20^. In our 3D model, the Mw7.2 MYG earthquake initiated spontaneously from the center of the MYG patch approximately 18 years following the Mw9.1 earthquake (Fig. 5h).

In contrast, the stress increased at a low rate (solid red line in Fig. 7b) following the Mw7.3 MYG earthquake during the later stage of the M ~ 9 earthquake cycle, and the entire MYG patch was locked when the afterslip from the Mw7.3 MYG earthquake occurred in the surrounding area (Fig. 7d).The Mw7.4 MYG earthquake occurred approximately 39 years following the Mw7.3 MYG earthquake. The difference between the stress rates of the two periods (Fig. 7a,b) was caused by the slip velocity distribution. Thus, the time interval between the M ~ 9 earthquake and the M ~ 7 MYG earthquake was equal to or shorter than the recurrence intervals between the M ~ 7 MYG earthquakes in the later stage of the M ~ 9 earthquake cycle.

We do not emphasize that the MYG earthquake will occur 18 years following the Mw9.1 earthquake as shown in the model in Fig. 5h. Rather, we suggest that the MYG earthquake will occur repeatedly even during the early stage of the M ~ 9 earthquake cycle. Thus, we should be careful and prepare for a future M ~ 7 earthquake in the Miyagi-ken-Oki segment shortly following the occurrence of the 2011 great earthquake.

## Methods

### Numerical calculation

Numerical simulations were based on the method used by Nakata *et al.*^24^ and Ohtani *et al.*^22^. Seismic and aseismic events were modeled to represent the release of slip deficit or backslip^29^ that accumulates during interseismic periods^30^. The 3D geometry of the Pacific Plate^31^ was similar to that used by Ohtani *et al.*^22^. We discretized the subducting plate into small subfaults in which the length in the *x* direction (N21.5°E) varied with depth or strike and ranged from 1.0 km to 9.0 km. Hereafter, subscripts *i* and *j* denote subfaults modeled for the simulation. The time derivative of shear stress τ on the *i*-th subfault owing to slip at the *j*-th subfault is calculated as


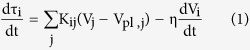


where K_ij_ is the stress increment on a subfault *i* owing to unit slip on a subfault *j*, V_j_ is the slip rate of the *j*-th subfault in the plate convergence direction, V_pl,j_ is the plate convergence rate of the *j*-th subfault, and η denotes the seismic radiation damping term^30^. We used η = 0.3G/2β,G is the rigidity (30 GPa)^25^; and β is the shear wave speed (3.27 km/s). We set the plate convergence rate as V_pl_ = 8.4 cm/yr^22^. Our methods differ from those of Ohtani *et al.*^22^ and Nakata *et al.*^24^ in terms of η in equation (1). We used a smaller value of the seismic radiation damping term to reproduce a shorter coseismic propagation time during the M ~ 9 earthquake.

Space-time variations in slip velocity are assumed to be an unstable slip with a frictional interface. Although we do not know the true friction law for the plate interface, we used a rate- and state-dependent friction law as an approximated mathematical model for large-scale frictional behavior on the plate interface^23^,^32^:


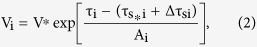



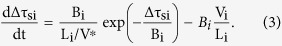


Equation (2) represents a fault constitutive law^33^ that determines the slip rate V_i_ for a given stress τ_i_ and a value of τ_si_ (=τ_s*i_ + Δτ_si_). If we set the reference velocity V* at a slip velocity level below which the slip is sufficiently slow to be negligible, the value of τ_si_ is the threshold level of stress τ_i_ required to cause significant slip velocity. Therefore, the value of τ_si_ is analogous to the “strength as a threshold”^33^, and we refer to it simply as “strength”. The parameter A (=aσ) controls the slip increase rate at which the stress reaches the strength. We did not treat the effective normal stress σ directly because its effect is difficult to separate from that of the frictional parameter a (and b, as will be described below) for natural earthquakes. V* was set to V_pl_ in the calculation below; here, τ_s*_ (=μ*σ) represents the steady state strength with V = V*, and Δτ_s_ is the variation in strength from the steady state.

Equation (3) is an aging law^32^,^34^. When strength is defined in this manner, equation (3) can be considered as an evolution law for strength change Δτ_s_, which varies depending on the prior slip history. The parameters B (=bσ) and L control strength recovery and slip weakening. For slip weakening, B and L determine primarily the amplitude of strength variation Δτ_s_ and slip weakening distance D_c_. These are the minimum characteristics of large-scale friction and are caused by various small-scale physical processes. From this perspective, frictional parameters A (=aσ), B (=bσ), and L in the rate- and state-dependent friction law are mathematical fitting parameters, which were assumed to be constant over the earthquake cycle in our simulations.

To solve equations (1)–(3), 2, 3, we removed the time derivative of stress dτ_i_/dt and obtained differential equations for slip rate V and strength Δτ_s_. Assuming the initial values given below, differential equations were solved with an adaptive time step fifth-order Runge–Kutta algorithm^35^. For the initial conditions, slip velocity V was assumed to be uniform at 0.9 V_pl_, and strength was calculated as τ_s*i_ + Δτ_si_ = Δτ_si_ = −B_i_ln(V_i_/V_pl_). For the boundary conditions, slip velocity was assumed to be constant and equal to V_pl_ outside the model region.

### Frictional parameters

The frictional heterogeneity at the shallower part of the Miyagi-ken-Oki region was assumed to be slightly stronger (L = 0.20 m, A–B = −0.181 MPa) than the surrounding area (L = 0.30 m, A–B = −0.10 MPa) over a wide range of 150–170 km long along the strike and 8–22 km in depth. The extent of M ~ 9 rupture area was controlled by the larger value of L, which means larger slip weakening distance and fracture energy, in the shallower part of the Miyagi-ken-Oki region and in the surrounding background area of A–B < 0. In addition, we used five circular patches termed SHL1, SHL2, MYG, FKS, and IBK, as the past sources of the 1981, 2003, 1978 Miyagi-ken-Oki, and 1938 Fukushima-ken-Oki earthquakes and the 2011 Ibaraki-ken-Oki largest aftershock, respectively (Fig. 2).

## Additional Information

**How to cite this article**: Nakata, R. *et al.* Possible scenarios for occurrence of M ~ 7 interplate earthquakes prior to and following the 2011 Tohoku-Oki earthquake based on numerical simulation. *Sci. Rep.* **6**, 25704; doi: 10.1038/srep25704 (2016).

## Supplementary Material

Supplementary Information

## Figures and Tables

**Table 1:** Frictional parameter values shown in Fig. 2.

	Length along strike (km)	L (m)	A–B (MPa)	|a/b|
Background (A–B > 0)	480	13	0.100	1.15
Background (A–B < 0)	480	0.30	−0.100	0.88
Center of the M ~ 9 area (8–22 km depth)	150	0.20	−0.181	0.81
	Radius (km)			
Foreshock (SHL1)	24	0.068	−0.325	0.70
SHL2	20	0.068	−0.323	0.70
Miyagi-ken-Oki (MYG)	23	0.021	−0.285	0.72
Fukushima-ken-Oki (FKS)	20	0.021	−0.278	0.73
Ibaraki-ken-Oki (IBK)	18	0.021	−0.312	0.71
