# Supplementary material for: Possible scenarios for occurrence of M ~ 7 interplate earthquakes prior to and following the 2011 Tohoku-Oki earthquake based on numerical simulation
Source: Sci Rep. 2016 May 10;6:25704. doi: 10.1038/srep25704 (PMC4861985; doi:10.1038/srep25704)
Supplement: Supplementary Information [file srep25704-s1.pdf]

Supplementary information

Possible scenarios for occurrence of M~7 interplate earthquakes prior to and following the 2011 Tohoku-Oki earthquake based on numerical simulation

Ryoko Nakata[1]*, Takane Hori[1], Mamoru Hyodo[1], & Keisuke Ariyoshi[1]

[1]Research and Development Center for Earthquake and Tsunami, Japan Agency for Marine-Earth Science and Technology, 3173-25, Showa-machi, Kanazawa-ku, Yokohama, Kanagawa 236-0001, Japan

*nakatar@jamstec.go.jp

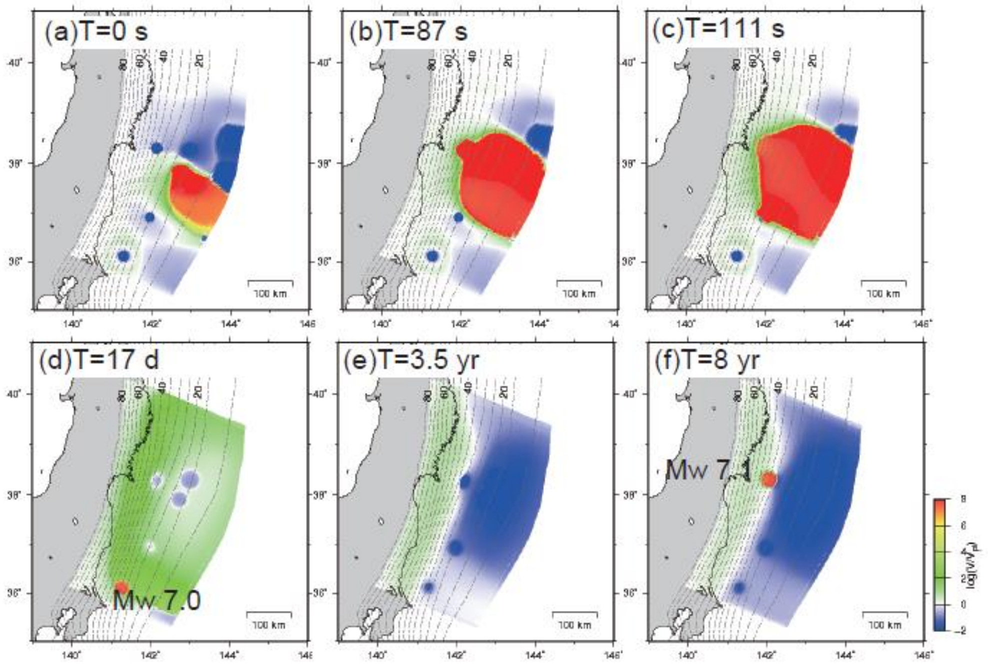

Figure S1. Slip velocity distribution normalized to the plate convergence rate from the initiation of the Mw9.2 earthquake to 8 years following the Mw9.2 earthquake, which was obtained using parameter sets shown in Table S1. Color scale is the same as that of Fig. 5. (a) Initiation of Mw9.2 earthquake. (b) Propagation of coseismic rupture of the Mw9.2 earthquake to the MYG patch. (c) Propagation of coseismic rupture of the Mw9.2 earthquake to the FKS patch. (d) Mw7.0 earthquake (aftershock) occurred at the IBK patch. (e) Continued afterslip. (f) Occurrence of Mw7.1 earthquake in the MYG patch. The maps were created by using Generic Mapping Tools software (GMT v4.5.12; http://gmt.soest.hawaii.edu/)[37].

Table S1. Frictional parameter values shown in Fig. 3b.

	Length along strike (km)	L (m)	A–B (MPa)	|a/b|
Background (A–B > 0)	480	13	0.100	1.15
Background (A–B < 0)	480	0.30	-0.100	0.88
Center of the M~9 area (10–22km depth)	170	0.20	-0.181	0.81
	Radius (km)	L (m)	A–B (MPa)	|a/b|
Foreshock (SHL1)	24	0.068	-0.325	0.70
SHL2	20	0.068	-0.323	0.70
Miyagi-ken-Oki (MYG)	23	0.021	-0.285	0.72
Fukushima-ken-Oki (FKS)	20	0.021	-0.290	0.72
Ibaraki-ken-Oki (IBK)	18	0.021	-0.3125	0.71

Table S2. Frictional parameter values shown in Fig. 3c.

	Length along strike (km)	L (m)	A–B (MPa)	|a/b|
Background (A–B > 0)	480	13	0.100	1.15
Background (A–B < 0)	480	0.30	-0.100	0.88
Center of the M~9 area (8–22 km depth)	165	0.20	-0.181	0.81
	Radius (km)	L (m)	A–B (MPa)	|a/b|
Foreshock (SHL1)	24	0.068	-0.325	0.70
SHL2	20	0.068	-0.323	0.70
Miyagi-ken-Oki (MYG)	23	0.021	-0.285	0.72
Fukushima-ken-Oki (FKS)	20	0.021	-0.287	0.72
Ibaraki-ken-Oki (IBK)	18	0.021	-0.312	0.71
